# Coasting: Model description, global sensitivity analysis, and scenario discovery

**DOI:** 10.1016/j.mex.2020.101145

**Published:** 2020-11-12

**Authors:** Jillian Student, Mark R. Kramer, Patrick Steinmann

**Affiliations:** a Environmental Policy Group, Wageningen University & Research, the Netherlands; b Environmental Systems Analysis Group, Wageningen University & Research, the Netherlands; c Information Technology Group, Wageningen University & Research, the Netherlands; d Biometris, Wageningen University & Research, the Netherlands

**Keywords:** Agent-based modelling, ODD+D, Global sensitivity analysis, Scenario discovery, Coasting model, ODD, Coastal tourism

## Abstract

This manuscript provides information for replicating the *Coasting* agent-based model presented in “Simulating emerging coastal tourism vulnerabilities: an agent-based modelling approach”. The model description follows the Overview, Design Concepts, and Details + Human Decision-making (ODD+D) protocol. Moreover, this paper includes implementation details on global sensitivity analysis and scenario discovery. Finally, we provide supplementary tables and figures for scenario discovery results not included in the main paper.

Highlights:

• Model description for simulating emerging environmental vulnerabilities in a coastal tourism context
• *Coasting*’s design facilitates model adaptations to other coastal tourism destinations
• Implementation details for applying global sensitivity analysis and scenario discovery to vulnerability assessments

## Graphical abstract

**Figure:**
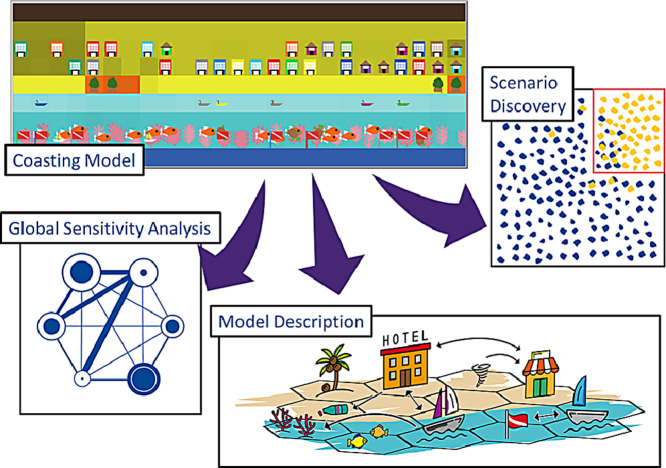

**Specifications Table**

**Table:** 

Subject Area:	Environmental Science
More specific subject area:	*Coastal tourism destination research*
Method name:	*Agent-based modelling, Overview, Design Concepts, and Details+ Human Decision-making (ODD+D) protocol D, global sensitivity analysis, scenario discovery*
Name and reference of original method:	Agent-based modelling Railsback, S., & Grimm, V. (2011). *Agent-Based and Individual-Based Modeling: A Practical Introduction*. Princeton; Oxford: Princeton University Press. doi: 10.2307/j.ctt7sns7 Overview, Design concepts, and Details (ODD) protocol Grimm, V., Berger, U., Bastiansen, F., Eliassen, S., Ginot, V., Giske, J., … DeAngelis, D. (2006). A standard protocol for describing individual-based and agent-based models. *Ecological Modelling*, 198(1–2), 115–126. Global sensitivity analysis Saltelli, A., Ratto, M., Andres, T., Campolongo, F., Cariboni, J., Gatelli, D., Saisana, M. & Tarantola S. (2008) *Global Sensitivity Analysis: The Primer.* John Wiley & Sons, Ltd. Scenario discovery Bryant, B. P., & Lempert, R. J. (2010). Thinking inside the box: a participatory, computer-assisted approach to scenario discovery. *Technological Forecasting and Social Change*, 77(1), 34–49. 10.1016/j.techfore.2009.08.002
Resource availability:	*Coasting model*https://harmoniqua.wur.nl/coastingmodel/ *NetLogo 6.0.4. Wilensky, U. (1999). NetLogo.* http://ccl.northwestern.edu/netlogo/*. Center for Connected Learning and Computer-Based Modeling, Northwestern University, Evanston, IL.* *SALib - Sensitivity Analysis Library in Python*https://salib.readthedocs.io/en/latest/# *Exploratory Modelling and Analysis (EMA) Workbench*https://emaworkbench.readthedocs.io/en/latest/ *PyNetLogo*https://pynetlogo.readthedocs.io/en/latest/

## Introduction

We present a detailed description of the *Coasting* simulation model [22]. The model endeavours to understand emerging vulnerabilities that tourism operators encounter; tourism operators are considered the most vulnerable of the coastal tourism sector as tourists have a higher adaptive capacity due to their ability to choose alternate locations, activities, timing (e.g. [10,^15^,24]). The model is made as part of a dynamic vulnerability approach [22], [23]]. We first describe the *Coasting* simulation using the ODD+D (Overview, Design Concepts and Details + Human Decision-making), then provide details about the global sensitivity analysis in the following section, and discuss our application of scenario discovery in the final section.

## Coasting ODD+D

This section describes the *Coasting* model. The simulation model was developed in NetLogo 6.0.4 [25]. The ODD (Overview, Design Concepts, and Details) follows the format of Grimm et al. [6] and Müller et al.’s [16] ODD+D extension including more information on human decision-making.

### Overview

#### Purpose

The intended audience is researchers and interested coastal tourism stakeholders. The general purpose is to explore emergent socio-ecological vulnerabilities occurring in coastal tourism settings and this version is instantiated for the island of Curaçao [22]. It also gives the opportunity to visualise different types of environmental change in the coast for stakeholders. This particular version of the model explores how vulnerabilities emerge over time due to unknown events (a proxy for many types of quick onset events), slowly developing sea-level rise (SLR), and locally-induced vulnerabilities (aggregated as pollution). The model also shows the interactions between tourism operators and their coastal environment.

The focus is on the tourism operators (supply side) as they have been identified as the most vulnerable (e.g. [10,^15^,24]). In general, operators want to have a sustainable business. This can mean financially sustainable and/or environmentally sustainable. In this version of the model, we do not model tourists as such, but rather their influence on the system. Tourists’ main influence is income (*tourism-returns*) mediated by operators’ inputs into the tourism product, and tourists’ contribution to pollution.

Several indicators are available to measure the emergence of socio-ecological vulnerability. The proxy for human vulnerability is the inability to survive as a business (having no reserves and going bankrupt), as well as not having enough reserves for allocating the recommended needed inputs for a sustainable business. Environmental vulnerability is measured by changes to environmental attractiveness of different spatial locations. It is divided into three regions: coastal (immediate land and water space), beach (inland location near coast), and nearshore (waters located near the coastline). Environmental attractiveness is made up of geospatial type, biodiversity, pollution level, and environmental degradation. Geospatial type and biodiversity contribute to environmental attractiveness whilst pollution levels and environmental degradation lower attractiveness.

#### Entities, state variables, and scales

This model includes two main types of agents: tourism operators on the one hand, and environmental resources (fish, sea turtles, coral reef, mangroves) on the other hand. Both are further divided into sub-types. Tourism operators (or operators for short) are the primary agents in this model. The model distinguishes five coastal operator types: hoteliers, beach vendors/operators (e.g. cafes, restaurant, and beach-based activities), nearshore operators (e.g. stand-up paddling, kayaking, surfing, and glass bottom boats), dive operators, and catamaran/boat operators. They seek to operate using certain environmental resources (e.g. beach, coral reef) under preferred environmental conditions (e.g. lack of pollution). Land-based operators are fixed to a certain location whereas water-based operators are mobile. Operators’ resources are a combination of the time, energy, expertise and finances that they put into their business. Different types of operators have similar input categories, but different input requirements for sustainable operations, different mobility, and different environment attribute preference. Table 1 shows the most important attributes of operators. Table 2 shows additional attributes that represent individual preferences of operators. The following symbols are used in the tables: { } for discrete values; [ ] for continuous values including the bounds; and ( ) for continuous values excluding the bounds. Operator agents are created during model initialisation. During a simulation, operators may go out of business, but no new operators are created dynamically.

**Table 1:** Main attributes of operator agents.

Variable name (as in model code)	Static or Dynamic	Range	Description
*resources*	dynamic	{0, 1, ...}	how many resources the operator has (proxy for time, experience, money); when an operator has 0 resources, the operator will go bankrupt
*needed-maintenance*	static*	{1, 2, 3, 4}	needed input resources for maintenance; value depends on operator type; *only changes with SLR interventions
*alloc-maintenance*	dynamic	{0, 1, ..., 7}	how much operator puts in for maintenance
*needed-tourism*	static	{1, ..., 5}	needed input resources for tourism product; value depends on operator type
*alloc-tourism*	dynamic	{0, 1, ..., 8}	how much operator puts in for tourism
*needed-environment*	static	{0, 1, 2}	needed input resources for environment; value depends on operator type
*alloc-environment*	dynamic	{0, 1, ..., 5}	how much operator puts in for environment
*needed-savings*	static	{0, 1}	recommended reservation of resources; value depends on operator type
*alloc-savings*	dynamic	{0, 1, ..., 4}	how much operator allocates to be saved
*delayed-maintenance*	dynamic	[0, 2]	how much operator is behind in maintenance; 0 means no delay 0‒1 slight delay, without penalty 1‒2 severe delay, incurs extra costs
*mobility*	static	{0, 1}	0 (immobile) for land-based operators; 1 (mobile) for water-based operators
*patch-here*	dynamic*	cell	(built-in NetLogo primitive) the cell where the operator is currently located; the operator can access all cell attributes of its current cell *Static for land-based operators
*base*	static	cell	for water-based operators only: base on land (as opposed to current cell); if this cell gets inundated, the operator goes out of business
*my-sites*	dynamic	set of cells	for water-based operators only: memory of cells where the operator has been
*max-possible-contribution*			not strictly a state variable, as it is not carried over between time steps; maximum amount of resources this operator can spend on all collaborative and individual actions together during this time step
*contribution*			not strictly a state variable either; actual contribution of this operator to the action under consideration

**Table 2:** Preference attributes of operator agents.

Variable name (as in model code)	Static or Dynamic	Range	Description
*default-maintenance*	static	{0, 1, ..., 5}	preferred allocation to maintenance; set to a value within 1 from *needed-maintenance*
*default-tourism*	static	{0, 1, ..., 6}	preferred allocation to tourism product; set to a value within 1 from *needed-tourism*
*default-environment*	static	{0, 1, 2, 3}	preferred allocation to environment; set to a value within 1 from *needed-environment*
*default-savings*	static	{0, 1, ..., 5}	preferred allocation to be saved; set to a value within 1 from *needed-savings*
*max-maintenance*	static*	{0, 1, ..., 6}	maximum allocation to maintenance; depends on operator type; *only changes with SLR interventions
*max-tourism*	static	{0, 1, ..., 10}	maximum allocation to tourism product; depends on operator type
*max-environment*	static	{0, 1, ..., 5}	maximum allocation to environment; depends on operator type
*max-savings*	static	{0, 1, ..., 5}	maximum allocation to be saved; depends on operator type
*wght-pos-maintenance*	static	{1, 2, 3}	relative weight for additional allocation to maintenance if sufficient resources
*wght-neg-maintenance*	static	{1, 2, 3}	relative weight for reduction of allocation to maintenance if insufficient resources
*wght-pos-tourism*	static	{1, 2, 3}	relative weight for additional allocation to tourism product if sufficient resources
*wght-neg-tourism*	static	{1, 2, 3}	relative weight for reduction of allocation to tourism product if insufficient resources
*wght-pos-environment*	static	{1, 2, 3}	relative weight for additional allocation to environment if sufficient resources
*wght-neg-environment*	static	{1, 2, 3}	relative weight for reduction of allocation to environment if insufficient resources
*wght-pos-savings*	static	{1, 2, 3}	relative weight for additional allocation to be saved if sufficient resources
*wght-neg-savings*	static	{1, 2, 3}	relative weight for reduction of allocation to be saved if insufficient resources

Operators record information on past collaborations with other operators. The model keeps a dynamic set of directed links for storing how successful attempted collaborations were. Table 3 shows the attributes of these links. When an operator goes out of business, its links are automatically destroyed. Otherwise, links stay in the model once they have been created. The model creates new links dynamically when operators (try to) collaborate with others, with whom they have no previously established link.

**Table 3:** Attributes of links between operators.

Variable name (as in model code)	Static or Dynamic	Range	Description
*end1*	static	operator	(built-in NetLogo primitive) observing operator
*end2*	static	operator	(built-in NetLogo primitive) observed operator from the perspective of the observing operator
*strength*	dynamic	[-1, 1]	how successful past collaborations were; 0 is neutral closer to 1, more successful closer to ‒1, less successful or free-riding

In the model, environmental resources are also considered agents; there are the following types: fish, sea turtles, coral reef, and mangroves. The former two are mobile while the latter are immobile. Table 4 shows the attributes of these environmental agents. Environmental resources can reproduce as well as die during a simulation, so their populations are truly dynamic.

**Table 4:** Attributes of environmental resources agents.

Variable name (as in model code)	Static or Dynamic	Range	Description
*health*	dynamic	[0, 1]	health indicator; at low health, the agent has a chance to die; at high health, it reproduces
*mobility*	static	{0, 1}	0 (immobile) for coral and mangroves; 1 (mobile) for fish and sea turtles
*patch-here*	synamic*	cell	(built-in NetLogo primitive) the cell where the agent is currently located; the agent can access all cell attributes of its current cell *Static for coral and mangroves

Each individual cell (patch in NetLogo) represents a part of the coastal system. The term cell(s) is used to describe individual spaces throughout this text. *Coasting* provides a simple spatial representation of the main coastal features (deep sea, shallow nearshore waters, coastal waters, coastal beach, prime nearshore land, nearshore land, subprime nearshore land, farther prime nearshore land, inland). The cells can be affected by pollution, environmental degradation, and SLR. Pollution is generated by operators' actions and is reflected in *pollution-level*. Environmental degradation is the manifestation of the negative impacts of a sudden event on a cell. SLR affects the elevation of cells. Table 5 shows the attributes of cells. Cells are never created or destroyed.

**Table 5:** Attributes of cells.

Variable name (as in model code)	Static or Dynamic	Range	Description
*geospatial-type*	static*	See Table 9	type of land or water (inland, coast, nearshore waters, deep sea) and quality of beach (sandy, rocky); *only changes with SLR
*elevation*	static*	[-50, 100]	level (in meters) above initial sea level; negative values for water indicate depth; *only changes by actions to cope with SLR
*pollution-level*	dynamic	[0, ∞)	from no pollution (0) to extremely polluted (≥1) in order not to lose pollution during dispersal, the value can exceed 1
*enviro-degradation*	dynamic	[0, 1]	level of degradation caused by sudden events; from no degradation (0), to extremely degraded (1)
attractiveness*	dynamic	[0, 1]	how attractive the environment is; based on attributes listed above and presence of environmental resources (biodiversity) *in current version referred to as patch-attractiveness

The model contains a limited number of global variables. Table 6 shows those global variables that cannot be reconstructed from other state variables. Apart from these, the model contains many global variables for summarising attribute values of agents and cells. These can be found under Observations in the Design Concepts section.

**Table 6:** Global variables.

Variable name (as in model code)	Static or Dynamic	Range	Description
*SLR-in-m*	dynamic	[0, ∞)	current sea level (in meters) above initial sea level
*ticks-since-sudden-event*	dynamic	{‒1, 0, 1, ...}	counter for determining times of sudden events; ‒1 means no sudden event has occurred
*total-num-collaborations*	dynamic	{0, 1, ...}	counter for all successful collaborative actions
*total-num-indiv-actions*	dynamic	{0, 1, ...}	counter for all successful individual actions
*lost-ops-due-to-SLR-land-based*	dynamic	{0, 1, ...}	total number of land-based operators that went out of business due to SLR
*lost-ops-due-to-SLR-water-based*	dynamic	{0, 1, ...}	total number of water-based operators that went out of business due to SLR

Spatial and temporal scales are approximate. The length and width of a cell are approximately 40 to 75 meters. Each time step represents approximately one and a half week. This is the average time a tourist stays in the area [3]. For this reason, we chose 35 time steps per year. The simulation runs for approximately 30 years of simulated time. Finally, Table 7 presents all model parameters and their corresponding ranges. The last entry of the table, *seed-for-random*, is a technical parameter to control reproducible randomness. It was included in global sensitivity analysis for quantifying the effect of stochastic aspects of the model.

**Table 7:** Model parameters.

Parameter	Range	Type
*tourism-returns*	[2, 5]	float
*revenue-limited?*	{False, True}	Boolean
*enviro-degradation-income-penalty*	{0, 1, …, 5}	integer
*maintenance-penalty*	[0, 3]	float
*pollution-penalty*	[0, 10]	float
*neighbor-pollution-penalty*	[0, 5]	float
*neighbor-pollution-threshold* (cells)	{4, 5, 6, 7, 8}	integer
*link-chance* (%)	[0, 10]	float
*links-to-my-base?*	{False, True}	Boolean
*negative-association*	[0.01, 0.25]	float
*positive-association*	[0.01, 0.20]	float
*geospatial-weight*	[0.0, 0.5]	float
*biodiversity-weight*	[0.0, 0.5]	float
*pollution-weight*	[0, 1]	float
*enviro-degradation-weight*	[0.0, 1.0]	float
*marine-life-sensitivity*	[0.10, 0.50]	float
*pollution-change*	[0.01, 0.50]	float
*pollution-diffusion-rate*	[0.01, 0.25]	float
*pollution-clean-up*	[0.01, 0.10]	float
*pollution-threshold*	[0.0, 0.5]	float
*cost-pollution*	{1, 2, …, 20}	integer
*SLR-increase* (mm/year)	[0, 50]	float
*linear-SLR?*	{False, True}	Boolean
*min-acceptable-elevation-above-SL*	[0.20, 1.00]	float
*increased-elevation*	[0.2, 1.0]	float
*erosion-loss*	[0.00, 0.25]	float
*cost-SLR*	{5, 6, …, 50}	integer
*sudden-event-interval*	{35, 36, …, 350}	integer
*patches-affected-sudden-event* (%)	[0, 10]	float
*enviro-deg-from-sudden-event*	[0.0, 1.0]	float
*acceptable-enviro-degradation*	[0.00, 0.50]	float
*cost-extreme-event*	{5, 6, …, 50}	integer
*sudden-event-persistence* (time steps)	{1, 2, …, 10}	integer
*seed-for-random*	32-bit integers	integer

#### Process overview and scheduling

*Set-up*

The environmental spatial setting and environmental resources’ abundance and locations are read from file. The five operator types are then set up. In the model version applied to Curaçao, each run has the same starting number and distribution of 75 simulated coastal tourism operators: 30 hotels, 10 beach operators, 20 dive operators, 5 boat operators, and 10 nearshore operators. First, the hotels and then the beach operators select an unoccupied land cell, preferably close to shore. Then the water-based operators are randomly assigned a land base, which they may share with a land-based operator. Then initial links are set up among operators. Initials link strengths between different types of operators are neutral, while for links among the same type a slightly negative link strength is initialised. Mobile operators are then given the opportunity to select a place in the sea. Finally, environmental resources’ health is initiated.

*At each time step*

**Fig. 1:**
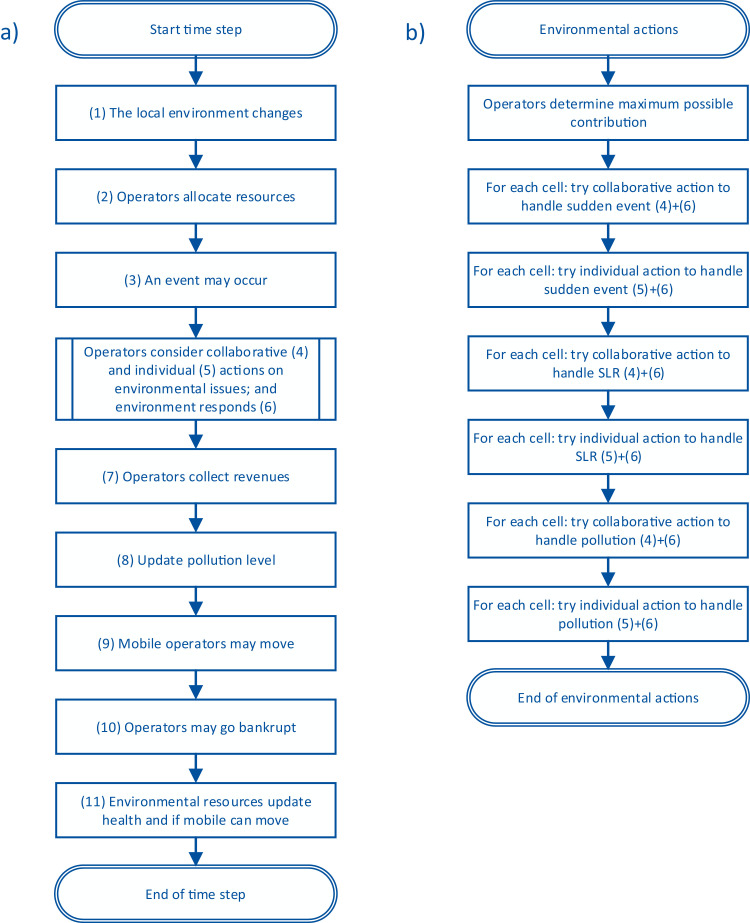
Flow chart of model steps.

The model performs the actions depicted in Fig. 1 each time step. Unless specified, for each action, all agents or cells perform the action consecutively in a random order (NetLogo's standard scheduling). First, the local environment changes: e.g. pollution disperses, environmental degradation may spread or decrease, and mobile environmental resources may move (1). Then operators determine how to plan their inputs for their operational budget in the “operators allocate resources" step (2). In step (3), an environmental event may occur: sea level rises or sudden event.

In steps (4), (5), and (6) operators decide whether to collaborate or act individually on environmental issues and the environment responds to the interventions (see Fig. 1b). First, all operators decide what is the maximum amount they have available for all environmental issues. Then, in random order, each cell is considered per type of event: first sudden events, then SLR, and then pollution. For each cell, the operators first consider if they are willing to collaborate on that issue (e.g. sudden events) (4). If there is sufficient support to collaborate on an issue, the operators act, and the environment responds to the intervention (6). Then another round is done for each cell for that same issue (e.g. sudden events) for individual action (5). If the issue has not been addressed by collaboration, individual operators who are directly or indirectly affected by that issue on the cell can decide to act alone. If the individual operator is willing and has enough resources to act on an issue, the environment responds to the intervention (6). This sequence repeats for the next type of environmental event (see Fig. 1b).

After the environmental action stage, operators’ expenditures are finalised and they collect their revenue based on their inputs in the tourism minus penalties for undesirable environmental conditions and maintenance delays in step (7). In the following step (8), operators update the pollution level of their current cell according to the balance between investments in the tourism product and the environment. If mobile operators find the environment conditions undesirable, they may decide to move to another cell in step (9). Then, operators without resources go bankrupt (10). Finally (11), environmental resources (fish, turtles, reef, and mangroves) update their health. Depending on their health, they may reproduce or die.

### Design concepts

In this presentation, we follow the terminology and order of concepts from Müller et al. [16]. The concepts “Individual prediction” and “Collectives” are not applicable in this case.

#### Theoretical and empirical background

The operators are modelled based on information acquired during literature review, (simulation-guided) interviews, simulation development, and serious games sessions. The level of aggregation depended on the means of access: literature data is typically at the destination (national level) or regional level; interviews (typically one on one, but on a few occasions with as many as five participants); focus group outputs (3 and 4 participants per session); and small group settings (3-8 participants) for simulation sessions.

All tourism operators have the same main input categories: maintenance, tourism product (marketing plus providing the tourism experience), and short-term environment (e.g. cleaning the beach, educating tourists not to damage coral). Operators need to look after their operational infrastructure (maintenance), otherwise they will eventually lose money (delayed effect), if they do not focus on tourists, then they will not be able to attract people to their business, if they do not look after their environmental situation, the surrounding environment will (slowly) get polluted. If they put more emphasis on their tourism product, this will have a negative impact on their environmental situation unless they also invest more in their environmental surroundings. In the game in general, people did not need to do more than the required amount for maintenance, but are penalised in the following steps when they do not keep it up. If they do not look after the environment it will degrade, if they put more into it, it is either compensation for more tourism activities or is a potential future investment in the environment.

The simulated operators are exposed to three different types of environmental events. The model focuses on locally-induced pollution as well as two types of globally driven change manifested at the destination: gradual onset through SLR on the one hand and sudden events that can be a proxy for immediate events such as hurricanes, coral bleaching event and other unknown and uncertain events on the other hand. Pollution results from insufficient environmental expenditures and changes the pollution level. Pollution can disperse to adjoining cells. Sudden events, i.e. uncertain and new events, can emerge in different spatial areas and their cause is unspecified. In the model, those situated immediately where this event occurs, are negatively affected, those in the perimeter are somewhat affected, those farther away are removed are unaffected unless the problem grows. Sudden events have a random chance of affecting a certain number of environmental cells. It has an amount of environmental degradation associated with it (severity) and this negatively affects environmental attractiveness. Sudden events also have a natural duration that can vary, which determines how low before it might go away if no one acts. SLR occurs at set rate per year, unless nonlinear SLR-rate is selected, then sea-level's rate increases (e.g. [2,^17^,20]). The loss of land can become more extreme through erosion.

Pollution, environmental degradation, and SLR influence environmental attractiveness. Environmental attractiveness is made up of geospatial type, biodiversity, absence of pollution, and lack of environmental degradation; this is in line with the potential implications of resource degradation effect on tourism (e.g. [4,8]). Pollution, environmental degradation, and SLR lower *attractiveness* of cells when present. Environmental attractiveness affects the earning potential of tourism operators.

In order to mitigate the negative effects of the environmental challenges on tourism operators, each environmental problem has an associated cost of dealing with them. Interventions directed to sudden events have a chance of removing environmental degradation. If the increased sea level (*SLR-in-m*) is greater than the elevation of a cell, that cell's geospatial type changes to coastal waters unless the operators intervene. For SLR, the intervention increases the elevation of the cell by a set amount; it also changes the geospatial-type to reinforced land, which has a lower geo-value than coastal land, contributes some pollution to neighbouring coastal cells, and increases the cost of maintenance per round for land-based operators whose cell is affected by the SLR intervention and for water-based operators who have their base on the affected cell. Interventions can remove chunks of pollution from *pollution-level*.

#### Individual decision-making

Tourism operators work with imperfect knowledge and individual weight preferences. Simulated operators use the weight preference for deciding on whether to collaborate, act alone, or do nothing. They use individual weight preferences for making input decisions and for reallocating resources when they have decided to contribute. They also use imperfect knowledge when deciding initial locations and for mobile operators, when they decide to move.

The general goal of the operators is to survive as a business in the coastal tourism setting. This means to have sufficient resources to meet the resource requirements to operate and not going out of business. This involves weighing of their individual expenditures (inputs), responding to what others are doing, and monitoring how their surrounding environment in changing.

Two levels of decision-making are included. At the first level, individuals decide on personal resource allocation, location, movement (if mobile), willingness to act alone, and willingness to collaborate. At the second level, if multiple individuals are willing to collaborate, they decide how much they will contribute to work on the problem together. Table 13 highlights the factors affecting their willingness.

Operators adapt their behaviour to both endogenous (network links to others, resource availability, action decisions) and exogenous variables (pollution levels, sea level rise, sudden events). The amount of available resources modify their resource allocation, willingness to act, the reallocation of resources if they have decided to act and have not exceeded the maximum possible contribution. In response to environmental threats, operators can do the following: collaborate, individually act, move away (if they are mobile), or do nothing. During each time step, they have an opportunity to decide on their resource allocation and whether and how to respond to events. However, events (sudden events, sea level rise, and pollution) play out on different temporal and spatial scales and will only trigger consideration when the degradation of a cell reaches a specified threshold that operators can perceive the event at a location (parameters: *acceptable-enviro-degradation, min-acceptable-elevation-above-SL, pollution-threshold*). This can result in multiple events occurring at the same time or in succession. Social norms play a role through the links, but culture is not explicitly modelled (see Table 13 for contributing factors). Spatial aspects play a role in whether an operator considers collaborating or acting individually in response to environmental change. They are more likely to act if their neighbouring area/environment is affected and most likely, if their own cell is affected.

Uncertainty is included in operators’ decision rules. For deciding whether to collaborate or act alone, several factors create a higher probability of them acting (see Table 13). They do not know whether others will collaborate, if they will contribute, and if so, how much. Moreover, actions do not guarantee that the expressed result is reached. Not all of the pollution may go away, the sudden event might persist, and SLR may pass the new elevation created to mitigate SLR.

Environmental resources also make simple decisions. If their marine sensitivity has been triggered (model parameter), mobile environmental resources will decide every time step whether or not they will move to another location within their geospatial context (nearshore waters). Their preference is to go to another site with a reef, or if there is no reef, to a spot in the water with lower pollution and degradation than their current location. Mobile environmental resources do not know for sure if the new location will be better than their current location nor the best possible location. Environmental resource numbers can increase (they reproduce) when their health gets above a certain threshold. Environmental resources can also die if there is too much pollution or degradation and their health gets too low.

#### Learning

Mobile operators and environmental agents can store previous locations, which are included as location options if they decide to move to another location in successive rounds. Operators’ decisions on whether and with whom to collaborate are influenced by previous attempts to collaborate. Operators modify their directed links to other operators based on whether collaborations were successful and whether the other operator contributed to that collaboration. This modifies with whom operators are willing to work in the future and whether they collaborate on particular issue.

#### Individual sensing

The costs for cognition and gathering information are not explicitly included in the model. The model assumes agents are aware of attributes of their current cell once a threshold has been met, and in some cases of neighbouring cells as well.

*Operators*

Operators can detect the presence of other operators in a cell they intend to occupy, during initialisation as well as at later time steps. They are aware of geospatial type and the presence of certain environmental resources. They sense pollution, SLR, and the effects of sudden events when these exceed their respective thresholds. They directly sense these threats when they affect their immediate cell, a neighbouring cell, or in the case of mobile operators, their base. Indirectly, they sense affected cells through their established links. Operators observe whether other operators are willing to collaborate and how much they contribute to acting on environmental threats. Mobile operators sense locations with potentially better environmental conditions (environmental attractiveness) in the same geospatial type (less pollution and environmental degradation, and in some cases larger presence of environmental resources); they can misjudge what a better location is for their tourism activities.

*Environmental agents*

Environmental resources sense pollution and environmental degradation over a sensitivity threshold. Like mobile operators, mobile environmental agents sense places with potentially better environmental conditions.

#### Interaction

Interactions among operators, environmental resources, and the environment are both direct and indirect.

*Operators to operators*

Operators can collaborate on environmental issues. Interaction is both indirect by attributes of cells and direct through links between operators. Operators’ actions affect pollution levels of an area negatively or positively, which influences income generated. Acting on environmental threats, whether individually or collectively, can improve environmental conditions and thereby improve operators’ revenue potential. Links are how operators record information on past (attempted) collaborations. Links with positive strength increase the willingness to collaborate on events of those linked to the affected operator(s), whereas negative links lower the chance of willingness to collaborate. Success of a collaboration depends on location, links, available resources, and willingness to collaborate. When a collaboration is successful, a positive link is created or the strength of an existing link becomes more positive. For an unsuccessful collaboration, a negative link is created or an existing link becomes more negative. Apart from the initialisation of links, the structure of the network is an emergent effect of collaborations.

*Operators to environmental resources*

There is only indirect interaction from operators to environmental resources. The operators can cause pollution to exist and persist as well as environmental degradation to persist in the cell occupied by environmental agents; this affects the health of environmental resource. This interaction depends on the location of the environmental threat and the actions of the operators.

*Operators to the environment*

The operators can cause pollution to exist and persist, as well as allow environmental degradation to persist in a cell. They can change the geospatial type of beach cells that will potentially become lower than sea level. This depends on the operators’ inputs as well as the actions on environmental threats.

*Environmental resources to environment*

Mangroves can help prevent beach geospatial type from turning into to water (add a fixed extra elevation buffer). In good health, environmental resource numbers can grow and spread to new cells. This depends on the sea level and sea level rise.

*Environment to operators*

Pollution and environmental degradation (can) negatively affect revenues and is dependent on the location.

#### Heterogeneity

The model includes heterogeneity at two levels. Operator types have many properties and decision-making processes in common, but some processes and preferences differ between operator types. Within each type of operators, different agents have their individual allocation strategies.

*Between operator types*

The operator types vary in required input levels for maintenance, tourism product (marketing and tourism experience), short-term environmental efforts, and savings. Moreover, they are heterogeneous in their preferences for locations and environmental resources as well as their mobility. Water-based operators are mobile while land-based operators are fixed to a specific location.

*Within operator types*

When resources exceed input requirements for the operator type, individual operators vary in the way they distribute the excess resources. Similarly, when resources are lower than input requirements, operators make different choices where to cut allocations. Operators also have individual preferences for the maximal amount to put towards each input category.

#### Stochasticity

*Order of agent and cell actions*

At any point in the model where all agents or cells of a specific type (or subset) have to perform a similar action, these agents or cells perform the action consecutively in a random order. This is the standard mechanism of NetLogo.

*Location*

Initially, operators choose their locations one by one. Each operator has a set of preferred locations determined by geospatial type and presence of environmental resources. An operator chooses with equal probability one location from the available preferred locations. For mobile operators and mobile marine life, if they decide to move, they choose with equal probability from the cells that meet their criteria. The elevation of each cell is uniformly distributed within a range specific to its geospatial type.

*Allocation of resources*

In case total resources of an operator do not exactly match preferred allocations to categories (maintenance, tourism, environment, savings), the operator adds or subtracts allocations influenced by individual weights preferences. This is achieved by drawing a uniformly random number up to the total weights, which is then compared to successive threshold values according to cumulative weights. When there are more resources than the preferred allocations, resources are added according to positive weights preferences until either the maximum amount per input category is reached or the total number of resources has been allocated. Conversely, if there are less resources than the operators’ preferred allocations, then the operators’ individual negative weights preferences remove resources until the number of allocated resources is equal to the available resources. When an operator decides to act on an event and needs to reallocate resources, the same mechanism is used.

*Binary probabilities*

Many binary choices in the model are controlled by a probability (either a parameter or a value computed specifically for that situation). In these cases, a number is drawn uniformly in the interval [0, 1) and compared to the probability specified. Examples are reproduction and death of environmental resources and development of sudden events’ effects. See Submodels in the Details section for specific cases.

#### Observations

Data for the following outputs is collected at different time steps for global sensitivity analysis and at each time step for scenario discovery. For global sensitivity analysis the output times are equivalent to 10-year intervals over a 30-year period: time steps 350, 700, and 1050.

The key results emerging from the runs are in Table 8 They focus on operators’ and ecological indicators of socio-ecological vulnerabilities.

**Table 8:** Model outputs.

Number of operators	Operators with sufficient revenue for operations	Operators short on revenue	Operators who have declared bankruptcy
*m-hotelops*	*m-hotelops-enough*	*m-hotelops-short*	*m-hotelops-bankrupted*
*m-beachops*	*m-beachops-enough*	*m-beachops-short*	*m-beachops-bankrupted*
*m-diveops*	*m-diveops-enough*	*m-diveops-short*	*m-diveops-bankrupted*
*m-boatops*	*m-boatops-enough*	*m-boatops-short*	*m-boatops-bankrupted*
*m-waterops*	*m-waterops-enough*	*m-waterops-short*	*m-waterops-bankrupted*
*m-all-ops*	*m-all-ops-enough*	*m-all-ops-short*	*m-all-ops-bankrupted*
**Number of operators with delayed maintenance**	**Average time with sufficient resources**	**Average time short on reserves**	
*m-hotelops-delayed-maint*	*m-hotelops-av-time-enough*	*m-hotelops-av-time-short*	
*m-beachops-delayed-maint*	*m-beachops-av-time-enough*	*m-beachops-av-time-short*	
*m-diveops-delayed-maint*	*m-diveops-av-time-enough*	*m-diveops-av-time-short*	
*m-boatops-delayed-maint*	*m-boatops-av-time-enough*	*m-boatops-av-time-short*	
*m-waterops-delayed-maint*	*m-waterops-av-time-enough*	*m-waterops-av-time-short*	
*m-all-ops-delayed-maint*	*m-all-ops-av-time-enough*	*m-all-ops-av-time-short*	
		*m-av-time-before-bankrupt*	
**Lost operators due to SLR**	**Number of environmental resources**	**Number of links**	**Number of environmental actions**
*lost-ops-due-to-*	*m-corals*	*m-total-links*	*total-num-collaborations*
*SLR-land-based*	*m-fishes*	*m-neutral-links*	*total-num-indiv-actions*
*lost-ops-due-to-*	*m-seaturtles*	*m-positive-links*	
*SLR-water-based*	*m-mangroves*	*m-negative-links*	
**Current environmental attractiveness**	**Cumulative average environmental attractiveness**	**Average pollution levels**	
*m-av-now-attr-beach*	*m-av-av-attr-beach*	*m-av-pollution-beach*	
*m-av-now-attr-coast*	*m-av-av-attr-coast*	*m-av-pollution-coast*	
*m-av-now-attr-nearshore*	*m-av-av-attr-nearshore*	*m-av-pollution-nearshore*	
*m-av-now-attr-area*	*m-av-av-attr-area*	*m-av-pollution-area*	

*Operator vulnerability indicators, number of*

• Operators with sufficient resources per agent type, and total for all operators
• Operators with insufficient resources per agent type, and total for all operators
• Operators in business (not bankrupt) per agent type, and total for all operators
• Operators who have gone bankrupt per agent type, and total for all operators
• Operators with delayed maintenance per agent type, and total for all operators
• Operators businesses lost due to SLR, land-based and water-based

*Duration operator vulnerability indicators, average time of*

• Operators with sufficient resources per agent type, and total for all operators
• Operators with insufficient resources per agent type, and total for all operators
• Before going bankrupt for all operators

*Environmental quality/Environment vulnerability indicators, average attractiveness (of cells)*

• Beach attractiveness, current and cumulative average
• Coast attractiveness, current and cumulative average
• Nearshore (water) attractiveness, current and cumulative average
• Overall attractiveness, current and cumulative average
• Average pollution level
• Number of fish, coral, mangroves, and sea turtles

*Actions taken to reduce environmental vulnerabilities, number of*

• Individual actions
• Collaborative actions
• Links (negative, positive, neutral, total)

### Details

#### Implementation details

The model was implemented in NetLogo 6.0.4. At initialisation, the model reads data for the environmental set-up from file. The model and the input file used for all experiments can be found at (https://harmoniqua.wur.nl/coastingmodel/). For experimentation with many parameter sets, one of the authors created a Java program that reads each parameter set from file, executes the model, and writes the corresponding simulation results to one or more other files. This program was used instead of NetLogo's Behavior Space for more specific control of parameter settings. Parameter sets for sensitivity analysis were generated by Python code using SALib [7]. For scenario discovery, the Exploratory Modelling & Analysis Workbench controls the NetLogo model directly.

#### Initialisation

The initial state of the model is based on the input file for the geospatial set-up and environmental resources for the coastal setting, in this case Curaçao. There is a difference between good beach, i.e. a sandy beach, and less desirable (sub-par) beach, i.e. a rocky beach. The first row is on the beachfront; the second row beach is farther away and access to the coast can be blocked by another operator who operates from the beachfront row.

The initialisation of the spatial area and environmental resources of the coastal system is always the same (except for some variation in elevation). Initial values of cell attributes *pollution-level* and *enviro-degradation* are zero. For each cell, the *elevation* is set to a uniform random number between bounds specific for their *geospatial-type* according to Table 9. After setting all other cell attributes and initialising environmental resources, all cells compute their environmental attractiveness as defined in Submodels of the Details section.

**Table 9:** Various values related to geospatial-type.

Code	*geospatial-type*	Lower boundfor elevation	Upper bound for elevation	*Geo* value
99	inland	10	10	0.00
75	faraway sub-par beach (3^rd^ row +)	1.5	3.5	0.15
70	faraway beach (3^rd^ row +)	1.5	3.5	0.20
66	sub-par beach (2^nd^ row)	5	5	0.35
65	sub-par beachfront (1^st^ row)	1	3	0.55
61	beach (2^nd^ row)	5	5	0.60
60	beachfront (1^st^ row)	1	3	0.80
59	elevated beach space	0	5	0.75
50	coastline inside high water mark	0.25	1	1.00
40	nearshore water's edge of coastline	0	0	1.00
20	nearshore coastal waters	-5	-15	0.75
10	deep sea	-50	-50	0.00

The number of each type of operator is the same in all runs (Table 11). Hotels and beach operators select a location with *geospatial-type* either beachfront or beach with good or sub-par sand quality. Hotels select first and choose any empty cell based on geospatial-type. Then, from the remaining cells, beach operators choose an empty cell of their preferred geospatial-type. Hence, land-based operators do not share the same cell with another hotel or beach operator. Next, water-based operators select their land base. They never share a base with another operator of the same type, but may share their base with operators of another type. Dive operators and boat operators prefer land base locations where a hotel or beach operator are located; dive operators can have a base at beachfront and beach locations while boat operators are limited to beachfront locations. Alternative locations for dive operators are any beachfront or beach cell. Alternative locations for boat operators are any beachfront location regardless of hotel or beach operator presence. When selecting a location for their land base, dive and boat operators have a probability of 0.1 or 0.2 respectively of opting for one of the alternative locations instead of one of their preferred locations. Nearshore operators select their land base at any beachfront location. If a water-based operator is not able to find a land base, they leave the system immediately. Preferences of operators are initialised according to operator types as defined in Table 11.

Operators that share their base (location for land-based operators) have mutual links from the start of the model if *links-to-my-base?* is true. If parameter *links-to-my-base?* is false, we skip these links. Other operators may have a link if their bases are neighbouring cells. Operators may have a link if their bases are neighbouring cells or they share their base while *links-to-my-base?* is false. Model parameter *link-chance* defines the probability percentage for a link being created in that case. Because links are directed, probabilities for links between two operators are independent. For all links created during initialisation, the *strength* is set to either –0.10 for links between operators of the same type (competitors) or 0 for all other links.

After links have been set up, water-based operators move to a water cell. Dive operators choose any nearshore coastal or nearshore water's edge cell, preferably with fish and at least medium coral coverage, else with fish and some corals, and otherwise a nearshore water's edge cell. Boat operators choose any nearshore coastal waters cell. Nearshore operators start their operations at the nearshore water's edge of the coastline.

The input text file consists of a grid of six-digit codes. Each individual code defines one model cell. Table 10 shows the codes used in this version of *Coasting*. The code can be expanded to allow for features that are more variable. The input text file determines the initial location and abundance of environmental resources. The initial health of environmental resources is set to 0.50.

**Table 10:** Initialisation environmental input data.

**Geospatial type**	
first two digits stands for land water feature	see code for geospatial type in Table 9
**Environmental resources**	
third digit denotes natural features:	8 mangroves 3 sea turtles and fish present 2 sea turtles present, but no fish 1 fish present, but no sea turtles 0 no fish or sea turtles
fourth digit denotes coral cover:	3 high coral cover/abundance 2 medium coral cover/abundance 1 low coral cover/abundance 0 no coral cover
fifth and sixth digits	not used in this version.

**Table 11:** Initialisation of operators.

	Hotel operators	Beach operators	Dive operators	Boat operators	Nearshore operators
number of this type	30	10	20	5	10
initial *resources*	12	7	7	7	3
*needed-maintenance*	4	1	2	2	1
*needed-tourism*	5	2	2	2	1
*needed-environment*	2	1	1	1	0
*needed-saving*	0	0	0	0	0
*default-maintenance*	max(0, *needed-maintenance* + dUniform(–1, 1))				
*default-tourism*	max(0, *needed-maintenance* + dUniform(–1, 1))				
*default-environment*	max(0, *needed-maintenance* + dUniform(–1, 1))				
*default-saving*	max(0, *needed-maintenance* + dUniform(–1, 1))				
*max-maintenance*	*needed-maintenance* + 2				
*max-tourism*	*needed-tourism* + 5				
*max-environment*	*needed-environment* + 3				
*max-saving*	*needed-saving* + 5				
*wght-pos-maintenance*	dUniform(1, 3)				
*wght-pos-tourism*	dUniform(1, 3)				
*wght-pos-environment*	dUniform(1, 3)				
*wght-pos-saving*	dUniform(1, 3)				
*wght-neg-maintenance*	dUniform(1, 3)				
*wght-neg-tourism*	dUniform(1, 3)				
*wght-neg-environment*	dUniform(1, 3)				
*wght-neg-saving*	dUniform(1, 3)				

Note: dUniform(*x, y*) is a discrete uniform distribution, giving a random whole number from *x* to *y* (both inclusive)

#### Input data

After initialisation, this model does not input further external data.

#### Submodels

Submodels were designed based on the empirical work in Barbados and Curaçao. Where possible, they closely follow the mechanisms presented in the simulation game *Coasting*.

The most important outputs of the model are environmental attractiveness, numbers of operators, and numbers of actions (collaborative and individual).

This section first describes the submodels related to *Environmental Attractiveness*: pollution, sudden events and environmental degradation, sea-level rise (SLR), biodiversity, and environmental attractiveness calculation. Then, it follows with the submodels related to allocation of *Resources and Revenues*, which lead to economic viability of operators: resource allocation, revenues, movement of operators, and bankruptcy. Finally, the *Environmental Actions* section describes the submodels related to operators’ actions and links: contributions to actions, decisions on actions, collaborative actions, and individual actions.

#### Environmental attractiveness.

***Pollution***

Pollution levels of individual cells change as a consequence of several main model steps. In step (1) pollution disperses, in steps (6) operators reduce pollution levels, and pollution can be generated in two ways: by actions against SLR in steps (6) or by economic activities in step (8).

Diffusion of pollution, in step (1), is controlled by the parameter *pollution-diffusion-rate*. All updates of the pollution properties of cells are processed simultaneously, technically by taking a copy before further processing. On land, pollution disperses to all neighbouring cells with the same or lower elevation; pollution will never move up-hill. At the coastline, the fraction of pollution that corresponds to the diffusion rate moves to one (random) neighbouring cell at the coastline. At sea, pollution disperses to all neighbouring cells except land cells that are at least one meter above sea level.

As a result of individual or collaborative actions to handle pollution in step (6) (see Fig. 1b) *pollution-level* is broken into chunks of 0.1. For each chunk, there is a 75% chance that the chunk will be removed if the pollution occurs on land, and a 50% chance that the chunk will be removed if the pollution occurs in the water. Technically, step (6) is performed during the corresponding action in step (4) for collaborative actions or step (5) for individual actions, as soon as the action goes through. Whenever a beach cell is elevated in an action to address SLR in step (6), the pollution level of neighbouring beach cells increases by 0.3.

In step (8), each operator changes the pollution level of their current cell according to the differences between needed and allocated resources for tourism and environment. Spending more resources on tourism than needed increases pollution, and spending more on environment than needed may decrease pollution. The balance between these is captured by a variable *D*:

$$D=max\left(A_{t}-N_{t},-1\right)-\left(A_{e}-N_{e}\right)$$

where *A*_t_ and *A*_e_ are the resources allocated to tourism and environment respectively, and *N*_t_ and *N*_e_ are the respective needed levels. If the difference between *A*_t_ – *N*_t_ is less than -1, then the value -1 is used instead to prevent too much advantage of lower tourism to getting rid of pollution.

From this value of *D*, the model computes the raw pollution effect *P_r_*

*P_r_* = *D* – 2 if *D* > 2
*P_r_* = *D* if *D* < 0
*P_r_* = 0 otherwise

For nearshore operators, their resources and impact are not as vast as the others, and the pollution effect is smaller. In this case the formulas are:

*P_r_* = *D*/2 if *A*_e_ = 0
*P_r_* = 1 – *A*_e_ if *A*_t_ ≥ 2 and *A*_e_ ≥ 1 and *A*_t_ > 2 × *A*_e_
*P_r_* = *A*_t_/2 – *A*_e_ if *A*_t_ ≥ 2 and *A*_e_ ≥ 1 and *A*_t_ *<* 2 × *A*_e_
*P_r_* = -0.5 if *A*_t_ = 1 and *A*_e_ = 1
*P_r_* = 0 otherwise

If the raw pollution effect is greater than zero, pollution increases at the rate determined by the parameter *pollution-change*:

$$pollution-level\leftarrow pollution-level+\left(pollution-change\times P_{r}\right)$$

If the raw pollution effect is less than zero, there is a chance that pollution will not decrease. The actual pollution effect *P* is either 0 or the absolute value of the raw pollution effect. Pollution decreases at the rate determined by the parameter *pollution − clean-up*:

$$\begin{array}{c} P=0\;\text{or}\;P=-P_{r}\;\text{both}\;\text{with}\;50\%\;\text{probability}\\ pollution-level\leftarrow pollution-level-\left(pollution-clean-up\times P\right) \end{array}$$

***Sudden events and environmental degradation***

Step (3) controls the occurrence and extent of sudden events. In steps (4) and (5) operators take action to handle the effects of sudden events. Sudden events take place at a fixed interval set by a parameter (*sudden-event-interval*). Another parameter (*patches-affected-sudden-event*) gives the chance (as a percentage) for each individual cell that environmental degradation due to the sudden event occurs on the cell. The effect of a sudden event on a cell is that its *enviro-degradation* is increased up to maximally 1 by the value of a parameter *enviro-deg-from-sudden-event*.

Environmental degradation (*enviro-degradation*) lasts for at least the duration of parameter *sudden-event-persistence* (time steps if no action is taken by operators). If the environmental degradation has not been resolved by collective or individual actions before that time (*sudden-event-persistence*), there is a 1/3 chance that environmental degradation will spread to one of the neighbouring cells, a 1/3 chance that it will remain the same, and a 1/3 chance that *enviro-degradation* disappear autonomously. In case the cell is affected by another sudden event in between, the time counter restarts counting.

As the result of a successful collaborative action in step (4) or individual action in step (5), there is a 50% chance that it will be resolved and *enviro-degradation* is reduced to 0 in step (6), otherwise *enviro-degradation* remains.

***Sea-level rise (SLR)***

At the start of each time step, the model computes the current sea level from SLR parameters: for linear SLR, *SLR-increase* is divided by 35 (SLR per year divided by time steps in a year); for non-linear SLR (parameter *linear-SLR?* is false), the *SLR-increase* is multiplied by 1.25 from time step 200, by 1.5 from time step 400, by 1.75 from time step 800, and by 2.0 from time step 1200 onwards. Then all land cells that border the sea are inspected for erosion. If a cell has an elevation lower than the current sea level plus erosion loss (parameter *erosion-loss*), that land cell becomes sea. When mangroves are present on a land cell, the cell is protected somewhat from erosion. Therefore, where mangroves are present, 0.2 meters is added to the elevation before deciding if the land becomes sea. When a land cell converts to sea, all operators that are located on that cell (or have their base there, in the case of mobile operators) go out of business.

As the result of a successful collaborative action in step (4) or individual action in step (5), the *elevation* of the cell goes up by an amount given as parameter *increased-elevation* and the *geospatial-type* changes to an altered beach type (*geo-type-elevated-space* value 59) in step (6). For land-based operators who are located on the cell or on a neighbouring cell maintenance obligations increase: *default-maintenance* and *needed-maintenance* both increase by 2. For water-based operators whose land base is located on the elevated cell maintenance obligations also increase: *default-maintenance* and *needed-maintenance* both increase by 1.

***Biodiversity***

Biodiversity is derived from the presence of environmental resources in a cell. In step (11), the model determines the *health* and thereby reproduction and death of the environmental resources. If *pollution-level* is greater than 0.25 or it exceeds *marine-life-sensitivity*, 0.01 is detracted from *health*. If *enviro-degradation* is greater than 0.10 or it exceeds *marine-life-sensitivity*, another 0.05 is detracted from *health*. Finally, if *enviro-degradation* is 0 and *pollution-level* is less than 0.02, *health* improves by 0.01. If health exceeds 1 or goes below 0, it is reset to 1 and 0, respectively.

If *health* decreases below 0.25, there is a 50% chance that the environmental resource dies. If their *health* is greater than 0.95, there is a 1% chance that they produce one offspring in their current cell, but only if this does not lead to more than 2 fish, 2 sea turtles, and 3 units of coral in that cell. The *health* of parent as well as offspring is reset to 0.5.

Mobile marine life (reef fish and sea turtles) select another nearshore water cell when their sensitivity threshold (*marine-life-sensitivity*) to environmental degradation or pollution level has been exceeded in step (1). They consider other ideal water cells that have at least one reef present and a nearshore water *geospatial-type*. They also consider alternative water cells that have either a lower *pollution-level* or lower *enviro-degradation* than *marine-life-sensitivity* and a nearshore water *geospatial-type.* If there are no alternative water cells, they consider any cells with nearshore water *geospatial-type* as alternative water cells. If there is at least one cell that meets the ideal criteria, there is a 95% chance that the marine life moves to one of the ideal cells. Otherwise marine life moves to one of the alternative cells.

***Environmental attractiveness calculation***

During step (1), after processing diffusion of pollution and the effects of SLR and sudden events, the model recomputes the environmental attractiveness of each of the cells. The attractiveness (*Att*) is given by:

$$Att=0.5+W_{\text{geo}}\times Geo+W_{\text{bio}}\times Bio-W_{\text{pol}}\times Pol-W_{\text{env}}\times Env$$

The resulting attractiveness is limited to the range [0, 1]. In this formula, *W*_geo_, *W*_bio_, *W*_pol_, and *W*_env_ are weighing parameters (resp. *geospatial-weight, biodiversity-weight, pollution-weight*, and *enviro-degradation-weight*), and *Geo, Bio, Pol*, and *Env* are the perceived aspects of attractiveness. The value of *Geo* is derived from cell attribute *geospatial-type* according to Table 9. The value of *Bio* (biodiversity) depends on presence of sea turtles, fish and coral according to Table 12. The values of *Pol* and *Env* are the corresponding cell properties *pollution-level* and *enviro-degradation*, where *Pol* is restricted maximally to 1.

**Table 12:** Values for perceived attractiveness Bio due to biodiversity.

Presence of mangroves/coral	Fish; sea turtles	Fish; no sea turtles	No fish; sea turtles	No fish; no sea turtles
Land: no mangroves	n/a	n/a	n/a	0.00
Land: mangroves	n/a	n/a	n/a	0.20
Sea: abundant coral	1.00	0.85	0.90	0.70
Sea: some coral	0.85	0.75	0.80	0.30
Sea: no coral	0.60	0.30	0.50	0.00

At the end of each time step, the model computes output variables *m-av-now-attr-beach, m-av-now-attr-coast, m-av-now-attr-nearshore*, and *m-av-now-attr-area* as the mean value of the attractiveness attributes of all cells with the respective type: beach (*geospatial-type* 60, 61, 65, 66); coast (*geospatial-type* 40, 50, 59) nearshore waters (*geospatial-type* 20), and overall for the three regions.

#### Resources and revenues.

***Resource allocation***

During step (2), all operators determine how they want to allocate their resources for their operations. Each operator sets the values of corresponding attributes *alloc-maintenance, alloc-tourism, alloc-environment*, and *alloc-savings*. In this process, operators use the respective individual preferences as defined in Table 1 and Table 2. The only other point in the model where allocations are changed, and these preferences used, is when operators have to redistribute resources in order to handle an event, during steps (4) and (5).

First, they set the allocations to their respective (individual) default allocations, so *alloc-tourism* is set to *default-tourism* and likewise for the other three allocations. Then, while their *resources* exceed total allocations and at least one allocation is below its maximum allocation (*max-tourism* etc.), they add one unit to one of the four allocations with relative chances defined by the corresponding weights for increasing (*wght-pos-tourism* etc.). Alternatively, when total allocations exceed their *resources* and at least one allocation is above zero, they subtract one unit from one of the four allocations with relative chances defined by the corresponding weights for decreasing (*wght-neg-tourism* etc.).

***Revenues***

Before processing operators’ revenues, in step (7), the model updates their *resources* according allocated expenses; *alloc-savings* is kept outside this update because savings are available as resources again in the next time step.

resources←resources−alloc-maintenance−alloc-tourism−alloc-environment

Next, the model updates the status of delayed maintenance for each operator and subtracts a penalty from their *resources* if they have delayed maintenance from the previous round. The amount of the penalty is the lesser value of parameter *maintenance-penalty* and the operator's *needed-maintenance*.

Then, it is determined whether the operator has a further delay or has caught up on their maintenance. If *alloc-maintenance* is equal to *needed-maintenance,* no change to *delayed-maintenance* occurs. When *alloc-maintenance* is less than *needed-maintenance* for that operator, *delayed-maintenance* increases by the difference between *needed-maintenance* and *alloc-maintenance*. If *alloc-maintenance* is greater than *needed-maintenance, delayed-maintenance* decreases by 0.5. If *delayed-maintenance* becomes less than 0, it is reset to 0.

Operator revenues is generated through investments in tourism modified by pollution, environmental degradation, and the location of the operator's tourism activities:

resources←round(resources+T−P−E−L)

Here *T* is the income from tourism and the other terms represent missed income due to pollution (*P*), environmental degradation (*E*), and a sub-optimal location (*L*).

By default, tourism revenues are equal to the allocation for tourism (*alloc-tourism, A*_t_) multiplied by the value of parameter *tourism-returns*. However, when parameter *revenue-limited?* is true, investing too much more in tourism than needed (*needed-tourism, N*_t_) will reduce the additional revenues.

T = A_t_ × tourism-returns if revenue-limited? is false
T = (N_t_ + 1) × tourism-returns + (A_t_ – N_t_ – 1) × (tourism-returns – 1) else

Missed income due to pollution (*P*) consists of two terms: pollution in the current cell itself, with a multiplication factor *pollution-penalty* (a parameter), and average pollution in neighbouring cells, with a multiplication factor *neighbor-pollution-penalty* (another parameter). Both terms are rounded to a whole number before adding the terms. If the number of neighbouring cells with pollution > 0.25 exceeds *neighbor-pollution-threshold* (a model parameter), then the average pollution of those cells counts, otherwise the average pollution of all eight neighbouring cells.

If *enviro-degradation* of the current cell is greater than 0, missed income due to environmental degradation (*E*) is equal to parameter *enviro-degradation-income-penalty*.

By default, missed income because of location (*L*) is 0. For mobile operators who are either boat or dive operators, *L* is 1 if there is no reef, sea turtles, nor reef present. For nearshore operators, *L* is always 0. For beach vendors/operator and hotels, *L* is 1 if their operation occurs on a cell with *geospatial-type* sub-par beach. Additionally, if they are located far from the beach, *L* is increased by 1; when less than two neighbouring cells have *geospatial-type* coast (50), the penalty is applied.

***Movement of operators***

Movement of operators has an indirect effect on their economic viability. During a time step, mobile operators (dive, boat, and nearshore operators) move after collecting revenues, so any effect on income takes place during the next time step. Dive operators only consider new cells with nearshore coastal water as the *geospatial-type*, nearshore operators only consider cells with nearshore water's edge as the *geospatial-type,* and boat operators consider both nearshore water's edge and nearshore coastal geospatial types. From these cells, operators select cells with *attractiveness* at least equal to the *attractiveness* of their current cell.

Operators maintain a set *my-sites* of previous cells they have been. Before adding new candidate cells, they prune *my-sites* to those previous cells that have an attractiveness of no less than their current site's attractiveness minus 0.10. Then, the operators add the selected candidate cells to *my-sites* and randomly select one of *my-sites* as the location to move their operations. Note that the current cell is always included in *my-sites*, so this action could result in no effective move.

***Bankruptcy***

In step (10) of the main process, any operators with no or negative *resources* goes bankrupt and ceases being part of the simulation.

At the end of each time step, the model computes output variables *m-x-ops, m-x-ops-enough, m-x-ops-short*, and *m-x-ops-bankrupted* for *x-ops* any type of operator (*hotelops, beachops, diveops, boatops, and waterops*) as well as *all-ops* (see Table 8 under Observations in the Design Concepts section). These are the major outputs regarding economic viability.

#### Environmental actions.

***Contributions to actions***

Before considering any actions, collaborative as well as individual actions, each operator determines how much resources they want to spend on actions by setting their *max-possible-contribution*. The value depends on their reserves, defined as their resources minus allocations for maintenance, tourism, and environment (this explicitly excludes allocations for savings). When reserves are larger than or equal to 3, the *max-possible-contribution* is set equal to the reserves. If reserves are greater than or equal to 0, the *max-possible-contribution* is set to 3. If reserves are less than 0, but *resources* are greater than or equal to the sum of *needed-maintenance, needed-tourism, needed-environment*, and *needed-saving*, then the *max-possible-contribution* is set to 3; if *resources* is less than this sum then *max-possible-contribution* is set to 2. Finally, m*ax-possible-contribution* is further modified by operator's preference for savings and available resources. If the operator's *wght-pos-saving* is greater than 2, 1 is subtracted from their *max-possible-contribution.* If *max-possible-contribution* is greater than *resources, max-possible-contribution* is set to 0.

When contributions to an environmental action are sufficient to fund the action, contributing operators (or the individual operator, in case of an individual action) change their allocation of resources. They change their resources based on whether they have enough unallocated resources available to cover their contribution. First, any unallocated resources are used. If that is insufficient to cover the contribution, the remaining amount is subtracted from *alloc-saving* if *alloc-saving* is large enough to cover the remaining contribution. If *alloc-saving* is still insufficient, the operator reduces their allocated resources to maintenance, tourism, the environment, and savings according to their weight preferences (*wght-neg-tourism* etc.) until the remainder of their contribution is covered. Then *max-possible-contribution* and *resources* are lowered by the amount they contributed to the environmental action, which limits their capacity to contribute to further environmental actions.

***Decisions on actions***

This model version handles events in the following order: sudden events, SLR, and pollution (see Fig. 1b). For each event, the model first processes collaborative actions cell by cell and then processes individual actions cell by cell. For each combination of action and cell, the model first checks if the vulnerability of the cell is above the vulnerability threshold for the current event. The vulnerability for pollution is equal to the current *pollution-level* of the cell, for SLR it is *min-acceptable-elevation-above-SL*, and for sudden events it is equal to the current *enviro-degradation* of the cell. Water cells are not vulnerable to SLR. The vulnerability threshold is given by parameters *pollution-threshold* for pollution and *acceptable-enviro-degradation* for sudden events. For SLR of land cells, the vulnerability threshold is equal to the cell's *elevation* minus the current sea level (*SLR-in-m*).

If the vulnerability of the cell is above the corresponding threshold, all operators determine their willingness to collaborate with other operators in trying to address the current environmental challenge on that cell. Willingness *W* to collaborate (or to take individual action) depends on multiple factors: whether the operator is directly affected (the operator is on that cell); whether the operator is indirectly affected (i.e. the affected cell is one of the cells surrounding this operator, or in the case of water-based operators, the cell is their land base); whether the operator is able to move away; and the positive and negative links to affected other operators. Let *Lp* be the number of links with *strength* > 0 from the current operator, and *Ln* the number of links with *strength* < 0 from the current operator.

Degrees of being affected:

*W* ← 0 if not affected
*W* ← 0.40 if directly affected, for collaborative actions
*W* ← 0.50 if directly affected, for individual actions
*W* ← 0.20 if indirectly affected
Influence of immobility:
*W* ← *W* + 0.20 if affected and not mobile
Influence of links, for collaborative actions only:
*W* ← *W* + 0.30 if *Lp* > *Ln*
*W* ← *W* + 0.35 if *Lp* < *Ln* and directly affected
*W* ← *W* + 0.10 if *Lp* < *Ln* and indirectly affected
*W* ← *W* + 0.45 if *Lp* = *Ln* and directly affected
*W* ← *W* + 0.20 if *Lp* = *Ln* and indirectly affected
*W* ← *W* + 0.15 with probability 0.5 if *Lp* = *Ln* and not affected

Operators are willing to collaborate or take individual action with probability *W* (*W* > 1 is interpreted as 100% probability). For individual actions, this probability determines whether the operator tries to take action. For collaborative actions, if at most one operator is willing to collaborate, no action takes place. Otherwise, the willing operators determine their contributions and update (or create) their mutual links.

For determining their contributions, all collaborating operators first decide on two maximum contributions *C1* and *C2. C1* is the maximum contribution that an operator would invest in the intervention. *C2* is the maximum contribution that an operator would invest considering the minimum (excess) resources the operator would have left after investing. *C1* and *C2* values take into account the following conditions: whether the operator has excess resources (their *resources* are more than total allocated or *alloc-savings* > 0); whether the operator has sufficient resources to meet their needed input allocations; whether the operator is directly or indirectly affected by the environmental challenge; and the operator's tendency to save (*wght-pos-saving* > 2 or not), see Table 13.

**Table 13:** Determination of contribution limits C1 and C2 for actions.

Conditions				Resulting values	
Excess resources?	resources at least what's needed?	saving tendency	affected?	*C1*	*C2*
yes		high	yes	*reserves* – 1	*reserves* – 1
yes		high	no	int(*reserves* / 2)	*reserves* – 2
yes		low	yes	*reserves*	*reserves*
yes		low	no	2	*reserves* – 1
no	yes		yes	3	int(*allocated* / 2)
no	yes		no	2	int(*allocated* / 2)
no	no		yes	2	*allocated* – 2
no	no		no	1	*allocated* – 1

Their actual contribution is further limited by their current *max-possible-contribution* and the amount needed to handle the specific event. To prevent one operator funding the entire collaboration, for collaborative actions the potential contribution of any single agent will always be less than the amount needed.

*Cp* = min(*Cx, Ce, C1, C2*) for individual actions
*Cp* = min(*Cx, Ce*–1, *C1, C2*) for collaborative actions
*Ce* = ceil(*pollution-level* / 0.1 × *cost-pollution*) for handling pollution (ceil is rounding up)
*Ce* = *cost-SLR* for handling sea-level rise
*Ce* = *cost-extreme-event* for handling environmental degradation
*Cx* = *max-possible-contribution*
*C1* and *C2* defined by Table 13 but reset to 0 if negative

Here, *pollution-level* is an attribute of the current cell, *max-possible-contribution* is an individual variable of the current operator, and *cost-pollution, cost-SLR*, and *cost-extreme-event* are model parameters.

In the resulting values columns of Table 13 *reserves* stands for *resources* minus allocations for maintenance, tourism, and environment (this explicitly excludes allocations for savings), while *allocated* stands for the total allocations to maintenance, tourism, environment, and savings.

For individual actions, *Cp* is the contribution. For collaborative actions, the willing operators determine their actual contributions from their *Cp* values by increasing their actual contribution from zero in rounds. In the first round, operators raise their contribution to 1, in the second round, to 2, and so on, as long as their contribution does not exceed their *Cp* and as long as the total contributions do not exceed the amount needed. The process stops when the amount needed is reached or no operators can increase their contribution anymore.

***Collaborative actions***

If the joint contributions of willing operators are less than the amount needed for the action, no action takes place. Otherwise, the willing operators adjust their allocations, *resources*, and *max-possible-contribution* accordingly. For the effects of actions, please refer to Pollution, Sudden Events, and Sea-level Rise at the start of this Submodels section.

In any case, whether collaboration leads to action or not, the attempted collaboration will influence link strengths between those operators willing to participate. Non-existing links are equivalent to links with *strength* 0, so before updating a link, the model will create it with *strength* 0 if it does not yet exist.

For updating the *strength* of the link from operator A to operator B, the model makes a number of choices depending on whether A is affected, A and/or B contributed at all, and whether B contributed more than A. Table 14 lists the updates of link *strength* for the different combinations. Values *positive* and *negative* relate to model parameters *positive-association* and *negative-association*, but the sign of *negative* has been reversed to render interpretation of the table more intuitive. If link *strength* exceeds its range (-1:1), it is reset to be (-1 or 1).

**Table 14:** Update to link strength from operator A to operator B for all combined choices.

Combined choices	Update if collaboration leads to action	Update if collaboration breaks down
A affected; *contrib.* B > *contrib.* A	0.25 × *positive*	1 × *positive*
A affected; *contrib.* A > 0; 0 < *contrib.* B ≤ *contrib.* A	1 × *negative*	0.5 × *positive*
A affected; *contrib.* A > 0; *contrib.* B = 0	2 × *negative*	0.5 × *negative*
A affected; *contrib.* A = 0; *contrib.* B = 0	1 × *negative*	0.5 × *negative*
A not affected; *contrib.* B > contrib. A	0.25 × *positive*	0.5 × *positive*
A not affected; *contrib.* A > 0; 0 < *contrib.* B ≤ *contrib.* A	0.5 × *negative*	0.25 × *positive*
A not affected; *contrib.* A > 0; *contrib.* B = 0	1 × *negative*	0.25 × *negative*
A not affected; *contrib.* A = 0; *contrib.* B = 0	0.5 × *negative*	0.25 × *negative*

***Individual actions***

Individual actions (5) follow a similar pattern to collaboration and use some of the same mechanisms. However, there are some slight differences. As indicated above, links have no influence on their willingness to act, and the willingness if they are immediately affected is slightly larger than for collaborative actions (0.50 instead of 0.40).

If an operator is willing to take individual action, they determine the amount of contribution required to act in the same way as operators do for collaboration. However, as the individual is the only one funding the environmental action, their contribution has to be equal to the amount needed. If their contribution is the greater than or equal to the amount needed, they perform the action and adjust their allocations, *resources*, and *max-possible-contribution* accordingly. For the effects of actions, please refer to “pollution”, “sudden events and environmental degradation”, and “sea-level rise” at the start of this Submodels section.

## Global sensitivity analysis

For global sensitivity analysis, we used SALib version 1.3.7 on Python 3.7.3 (Anaconda distribution 4.7.10). We generated parameter samples using the Saltelli method [19] saltelli.sample(...)implemented in SALib.sample with parameter calc_second-order=True and a fixed value for the seed parameter. The SALib problem description is a direct translation of the parameters table in Table 7. We translated Boolean parameters into a range [0, 1], where all values below 0.5 are interpreted as False and above 0.5 as True.

For analysis, we used the Sobol global sensitivity analysis method [21], which is implemented as sobol.analyze(...) in SALib.analyze, with parameter calc_second-order=True and a (different) fixed value for the seed parameter. The problem description parameter was identical to the one used for sampling.

SALib generates samples as a Pandas data frame with one column per parameter. Its analysis module expects a similar Pandas data frame with one column per model output. Passing this data to NetLogo, and storing the results, is not straightforward even when using NetLogo's BehaviorSpace tool. Moreover, for large numbers of runs, results should be stored immediately, rather than after all runs have been performed. Therefore, the second author has constructed a Java program that reads parameter values from a CSV file (previously generated with Python and SALib), controls NetLogo to run the model for these parameters, and writes selected outputs to another CSV file. This file is then imported back into Python for sensitivity analysis. The Java program can run subsets of the parameter file, which facilitates running sub-batches on several processors or even several computers concurrently. It also makes it easy to restart the runs after a power failure or an automatic restart of the computer.

For sensitivity analysis on *p* parameters, Saltelli sampling generates 2 × *n* × (*p*+1) samples, where *n* is the sampling rate. A minimum *n* of 1000 is advisable [9]. For testing the procedures, we first used *n*=100. Interestingly, the first test revealed one parameter as well as one output variable for which global sensitivity was 0 throughout. Both turned out to be (trivial) coding errors in the model, which were corrected before the full analysis. This highlights that sensitivity analysis can also be useful for model verification

The model has 34 parameters, so with *p*=34 and *n*=1000, this results in 70,000 simulation runs. For low sample sizes, approximation errors can yield sensitivity values slightly outside the theoretical range from 0 to 1. Analysis of the first 70,000 runs showed many negative values, which indicates that more samples were needed. The sensitivity results of a second batch of 70,000 runs differed considerably from the corresponding result of the first batch. Therefore, we extended to number of samples to *n*=10,000 (and hence 700,000 runs). We consider this number sufficient, as the results both converged, and gave sensitivity values we considered plausible. Following Jaxa-Rozen and Kwakkel [9], we did not perform replications of individual parameter combinations, instead accounting for stochasticity by including the *seed-for-random* technical model parameter in the global sensitivity analysis.

## Scenario discovery

Scenario discovery follows a three-step process [1]: data generation, identification of outcomes of interest, and rule induction. In the following, we present our method and choices for these three steps.

### Data generation & processing

We generated the scenario discovery data separately after the global sensitivity analysis in order to find the input parameter ranges that contribute to both economic and ecological vulnerabilities. Sensitivity analysis and scenario discovery are closely related model exploration methods, and it would principally be possible to re-use existing data from one for the other. Although this requires prior specification of the data requirements for both analyses during experimental design, it could generate substantial savings in terms of computation time. The data for scenario discovery was generated using the Exploratory Modelling & Analysis Workbench [11], which includes a PyNetLogo-based connector [9] for controlling NetLogo experiments. The Workbench uses SALib [7] to generate random samples of parameter distributions using a variety of sampling methods. We chose the Latin Hypercube method [13] as implemented in SALib. Model input parameters were sampled from the uncertainty ranges given in Table 7.

Based on expert feedback, we identified and recorded the most interesting model outcomes - the numbers of the various tourism-related businesses (hotels, beach, dive, boat, and nearshore operators), and the attractiveness of the various coastal zones (beach, coast, nearshore). Furthermore, the individual and collaborative actions were recorded to study (collective) response to climate change vulnerabilities.

We conducted 4000 simulation experiments (i.e. unique input parameter combinations) with the *Coasting* model, replicating each experiment 30 times to account for stochastic aspects of the model. These numbers were chosen as a balance between computational cost and coverage of the uncertainty space. The model was run for 1050 time steps, representing 30 years of future system behaviour in response to uncertain climate change. The outcomes of interest were recorded for every time step in every run.

After generating the experimental data, we processed it to make later analysis easier. NetLogo and the Workbench use different terminology for model time, requiring manual renaming of one experiment. We also averaged over the 30 replications of each experiment to reduce stochastic influence on the outcomes. This work was performed in NumPy [18] and Pandas [14].

### Identifying outcomes of interest

Scenario discovery seeks to identify the conditions, or ranges of input parameters, under which unacceptable outcomes may occur. For the *Coasting* model, we defined two basic scenarios which could be considered unacceptable. *Ecological Failure* represents a >25% drop in environmental attractiveness, while *Economic Failure* comprises a 75% drop in businesses in operation. We also studied a third scenario, *Combined Failure*, in which both basic failures occur. To evaluate which experiments fulfilled the scenario conditions, we compared the first and last values for each experiment's *m-av-now-attr-area* (for *Ecological Failure*) and *m-all-ops* (for *Economic Failure*) outcomes. If the last value exceeded the described thresholds vis-a-vis its initial value, we considered the experiment (and its input parameter combination) of interest for scenario discovery.

### Rule induction

We used the Workbench to perform rule induction, choosing the Patient Rule Induction Method (PRIM) [5] as our rule induction algorithm. We opted for the improved version of PRIM with a lenient objective function implemented in the Workbench [12].

Scenario discovery is an interactive process requiring a number of analyst decisions. These mainly revolve around trade-offs between three main metrics: coverage, density, and interpretability. Coverage represents how many of the decision-relevant (i.e. unacceptable) futures are included in the induced scenario region. This characteristic should be maximised to reduce false negatives (decision-relevant input parameter sets outside the region). Density captures the ratio of decision-relevant to irrelevant futures in the region, which should also be maximised to avoid false positives (decision-irrelevant inputs inside the region). Interpretability describes which dimensions the region has been restricted to, i.e. the more parameter dimensions, the more influencing input parameters the output has. To ensure the induced region is comprehensible and useful, this value should be minimised. As a general rule, we opted for higher coverage of the scenario regions, increasing the number of false positives, and accepted a penalty on density. We also tried to keep the number of restricted dimensions low. This proved difficult, as successively discovered boxes for the same scenario were not always restricted along the same input dimensions. In the following, we present the relevant parameter ranges for both basic scenarios, which were not discussed in Student et al. [22].

### Results

**Fig. 2:**
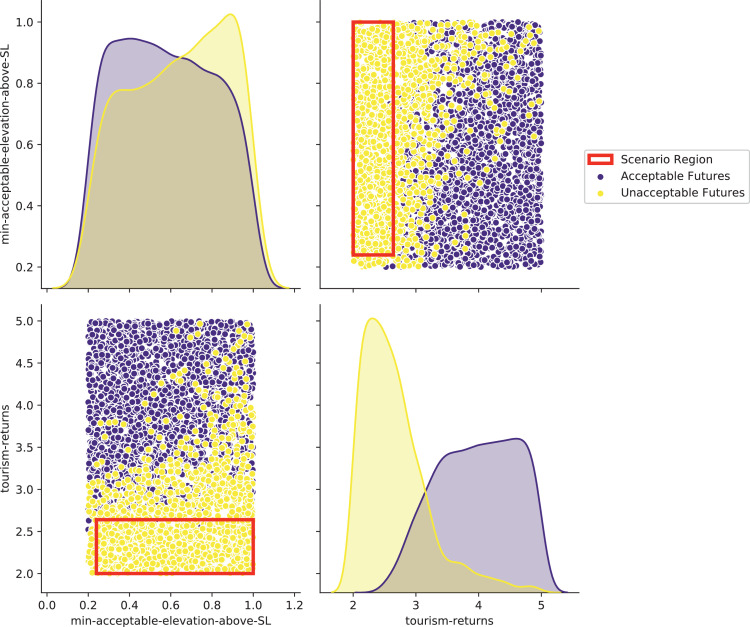
Economic failures PRIM box 1.

**Fig. 3:**
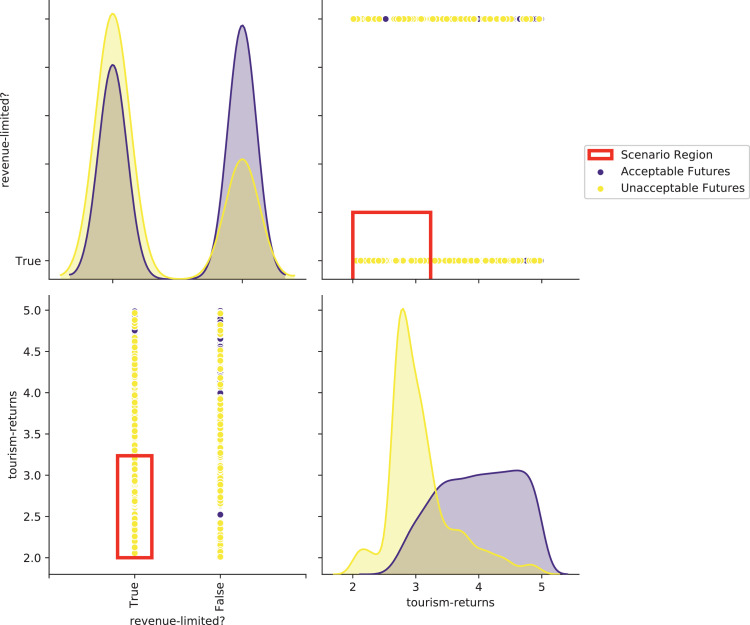
Economic failures PRIM box 2.

**Fig. 4:**
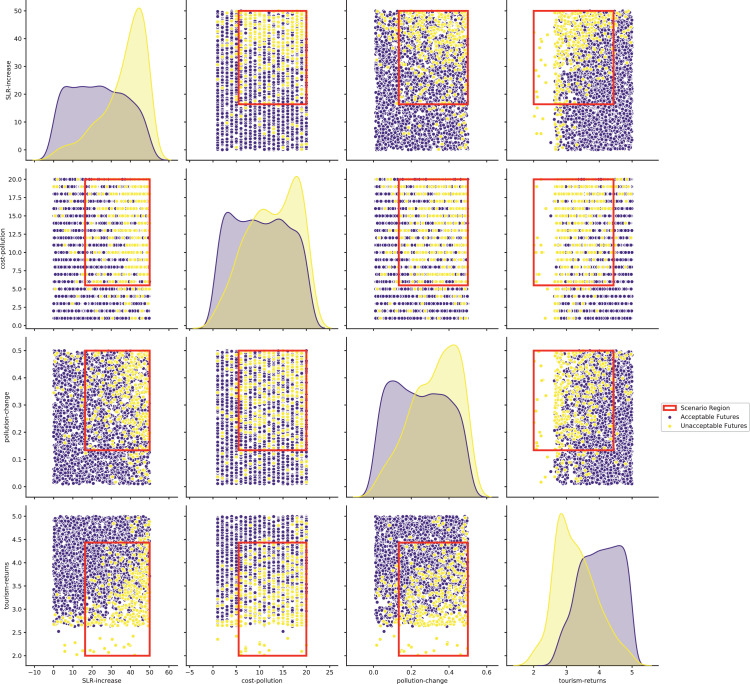
Economic failures PRIM box 3.

Due to space constraints, we could not present the results for *Economic Failure* and *Ecological Failure* in the main submission. For completeness, we present and briefly discuss those results here. *Economic Failure* of the system is largely driven by low returns from tourism—it is the dominant input parameter in all three identified PRIM boxes constituting the scenario region of the input space (see Figs. 2–4). In particular, almost 90% of all experiments which exhibited a collapse of the economic sector had a *tourism-returns* value of ~3.3 or lower (i.e. that high returns are necessary for economic success, see Table 15). Secondary predictors are *min-acceptable-elevation-above-SL*, the existence of a revenue limit (*revenue-limited?*), *pollution-change*, the *cost-pollution*, and *SLR-increase* (see Table 15 and Figs. 3–4).

**Table 15:** PRIM box information for economic failure.

**PRIM box**			**Input parameters** [default range]					
Number	Coverage	Density	*tourism-returns* [2, 5]	*min-acceptable-elevation-above-SL* [0.2, 1]	*revenue-limited?* {False, True}	*pollution-change* [0.01, 0.5]	*cost-pollution* [1, 20]	*SLR-increase* [0, 50]
1	54 %	98 %	[2, 2.6]	[0.24, 1]				
2	23 %	78 %	[2, 3.2]		{True}			
3	16 %	33 %	[2, 4.4]			[0.13, 0.5]	[5.5, 20]	[16, 50]

**Fig. 5:**
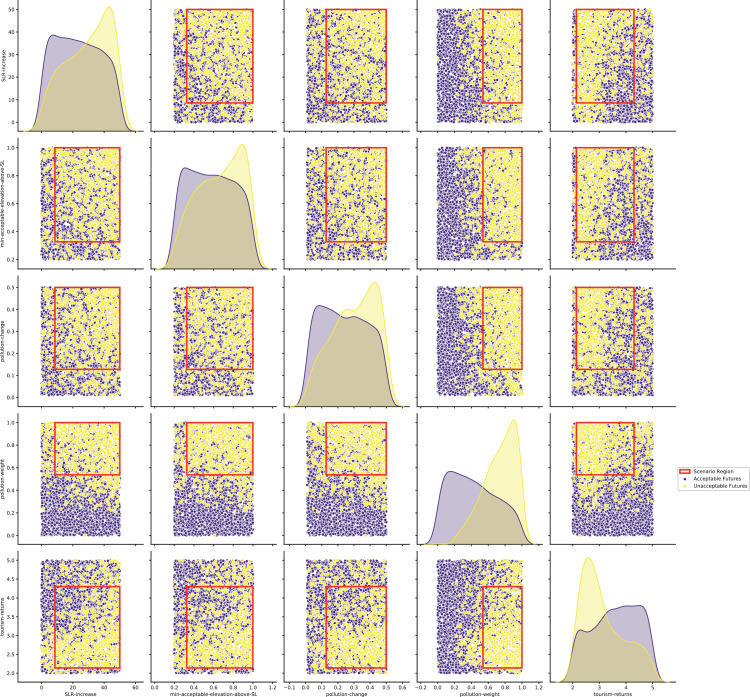
Ecological failures PRIM box 1; y-axis (Top to bottom) & x-axis (Left to right): SLR-increase, min-acceptable-elevation-above-SL, pollution-change, pollution-weight, tourism-returns.

**Fig. 6:**
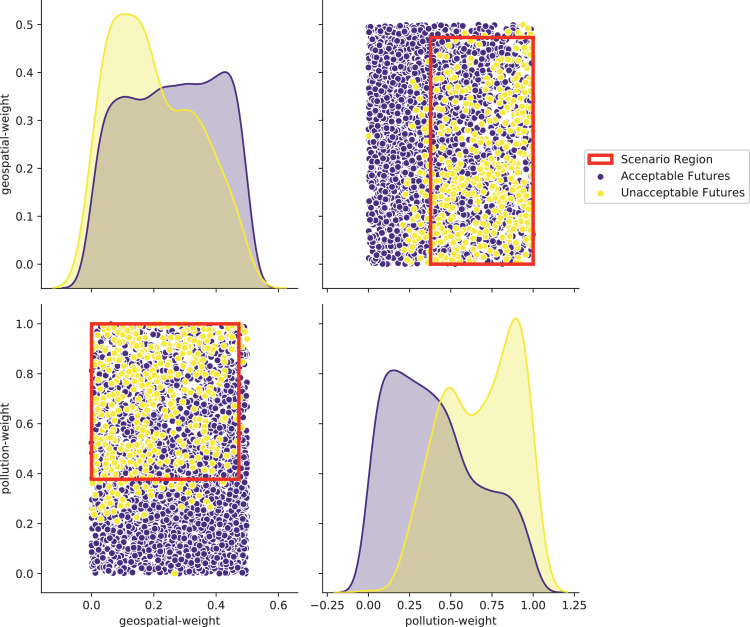
Ecological failures PRIM box 2.

*Ecological Failure* is largely driven by the actors’ pollution behaviour, perception thereof, and sea level rise (SLR). *Pollution-weight* is the dominant parameter for the ecological scenario region in both PRIM boxes (see Figs. 5–6). Secondary predictors are *tourism-returns, geospatial-weight, pollution-change, min-acceptable-elevation-above-SL*, and *SLR-increase* (Table 16).

**Table 16:** PRIM box information for ecological failure.

**PRIM box**			**Input parameters** [Default range]					
Number	Coverage	Density	*SLR-increase* [0, 50]	*min-acceptable-elevation-above-SL* [0.2, 1]	*pollution-change* [0.01, 0.5]	*pollution-weight* [0, 1]	*tourism-returns* [2, 5]	*geospatial-weight* [0, 0.5]
1	50%	63%	[8.6, 50]	[0.33, 1]	[0.13, 0.5]	[0.54, 1]	[2.1, 4.3]	
2	43%	24%				[0.38, 1]		[0.00012, 0.47]

## Declaration of Competing Interest

The authors have no conflicts of interest to declare.
